# Scrofulous Abscess of the Neck, with Perforation of the Jugular Vein; Death

**Published:** 1844-08-10

**Authors:** 


*Scrofulous abscess of the neck, with perforation of the jugular vein: death.*

A child, five years of age, on recovering from scarlatina, suffered an attack of inflammation and swelling in a cluster of subcutaneous glands situated on the right side of the neck. The glands suppurated. Fluctuation became distinct. A certain tremor, which was perceptible by the hand, and noise which could be heard by the ear applied to the abscess, were held suspicious symptoms, and much hesitation was therefore felt in puncturing the tumour; in fact, this was not done until after a consultation held with a regimental surgeon, who approved of the measure. The abscess was punctured, but immediately an ample stream of blood revealed the true nature of the mischief. The blood at first had a dirty red colour, undoubtedly from the admixture of pus; but before it could be arrested it appeared quite pure. The puncture was forthwith closed, and gentle pressure maintained by means of a bandage; but this was scarcely secured when the patient expired.

On an examination after death it was found that the external jugular vein was perforated like a sieve in a space three quarters of an inch in length, and that the parts of the vessel above and below this portion were discoloured and soft.

The abscess, which was regarded as metastatic in its nature, had, in fact, extended to the walls of the vein which lay over it, and perforated them.—*Dr. Hoffman, in Casper's Wochenschrift.*

[Our readers, on perusing the above interesting case, will all remember one almost precisely similar to it which occurred in the practice of a distinguished metropolitan hospital surgeon, some two years ago. The sole difference between the two cases was, that whilst in Dr. Hoffmann's the *vein* was perforated, in the one alluded to it was the *artery* that was ulcerated.]—*Ed. Med. Gaz.*

